# Single Crystal Diamond Needle as Point Electron Source

**DOI:** 10.1038/srep35260

**Published:** 2016-10-12

**Authors:** Victor I. Kleshch, Stephen T. Purcell, Alexander N. Obraztsov

**Affiliations:** 1 Department of Physics, M.V. Lomonosov Moscow State University, Moscow 119991, Russia; 2 Institut Lumière Matière, Université Claude Bernard Lyon 1 et CNRS, Villeurbanne 69622, France; 3 Department of Physics and Mathematics, University of Eastern Finland, Joensuu 80101, Finland

## Abstract

Diamond has been considered to be one of the most attractive materials for cold-cathode applications during past two decades. However, its real application is hampered by the necessity to provide appropriate amount and transport of electrons to emitter surface which is usually achieved by using nanometer size or highly defective crystallites having much lower physical characteristics than the ideal diamond. Here, for the first time the use of single crystal diamond emitter with high aspect ratio as a point electron source is reported. Single crystal diamond needles were obtained by selective oxidation of polycrystalline diamond films produced by plasma enhanced chemical vapor deposition. Field emission currents and total electron energy distributions were measured for individual diamond needles as functions of extraction voltage and temperature. The needles demonstrate current saturation phenomenon and sensitivity of emission to temperature. The analysis of the voltage drops measured via electron energy analyzer shows that the conduction is provided by the surface of the diamond needles and is governed by Poole-Frenkel transport mechanism with characteristic trap energy of 0.2–0.3 eV. The temperature-sensitive FE characteristics of the diamond needles are of great interest for production of the point electron beam sources and sensors for vacuum electronics.

Field electron emission (FE) from diamond has been a matter of intense research motivated originally by the possibility for realization of negative electron affinity (NEA) on certain diamond surfaces^1^,^2^. Potentially, electrons could be emitted from the flat NEA surface under very low electric fields which would eliminate the need for sharp tips that are needed for Fowler-Nordheim tunneling (see e.g. ref. 3). However, even if NEA were achieved on a suitable sample, the electrical conductivity of intrinsic wide band gap diamond is too low to allow sustained emission which would need electron transport through the bulk to the emitting sites on the surface. This can be circumvented in principle by doping or by introducing defects into the diamond structure. At the same time incorporation of high amount of defects will eliminate conditions necessary for NEA of the diamond surface and additionally will lower the excellent material characteristics of diamond bulk (e.g. high thermal conductivity, chemical inertness, mechanical stability) which are of special interest to provide high reliability and robustness of the FE cathodes. Therefore, in spite of extensive efforts made since 1990s, the development of FE cathodes with the use of doped^4^,^5^,^6^ or defective^7^,^8^,^9^ diamond have not led to the promised applications of flat diamond films in vacuum electronics. Much less work was done on microtip diamond FE cathodes, since their formation process is much more complicated and costly. The electron transport for a microtip cathode can be provided by the surface states in the diamond crystalline structure^10^. Simultaneously, such emitter could keep outstanding thermal and mechanical diamond properties in defect-free bulk and, potentially, negative or low positive electron affinity on its apex. Diamond microtips were made previously for example by reactive ion etching of polycrystalline diamond film^10^. This relatively simple method provides polycrystalline diamond emitters in the shape of microcones which demonstrate metallic type of electron emission with linear Fowler-Nordheim (FN) plots^11^. It has been revealed that emission properties in this case are determined by a conducting amorphous (i.e. essentially non-diamond) carbon layer on the emitter surface^12^.

In this paper we present a study of field emission from the single-crystal diamond needles produced by thermal oxidation of polycrystalline CVD diamond films^13^,^14^. Our previous studies^15^ revealed that the needles having high aspect ratios demonstrate temperature-sensitive highly nonlinear FN plots typical for semiconducting emitters^16^. Here, the measured FE characteristics were used to examine carrier transport mechanism in the diamond needles and reveal possibilities for their practical applications as a point source of electrons.

## Results

**Figure 1:**
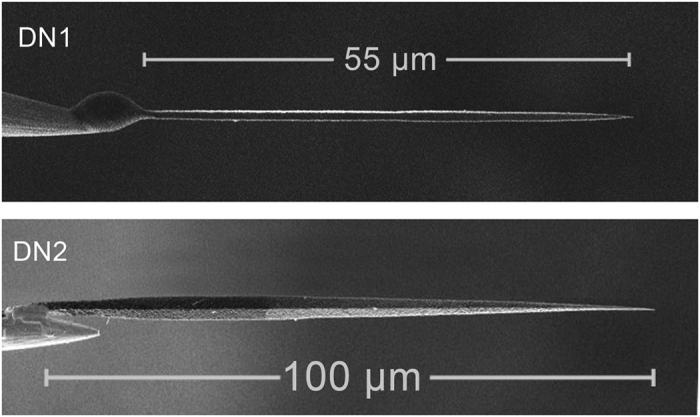
SEM images of two diamond needles DN1 (length 55 μm, max. width 0.85 μm) and DN2 (length 100 μm, max. width 3 μm) mounted on tungsten tips.

**Figure 2:**
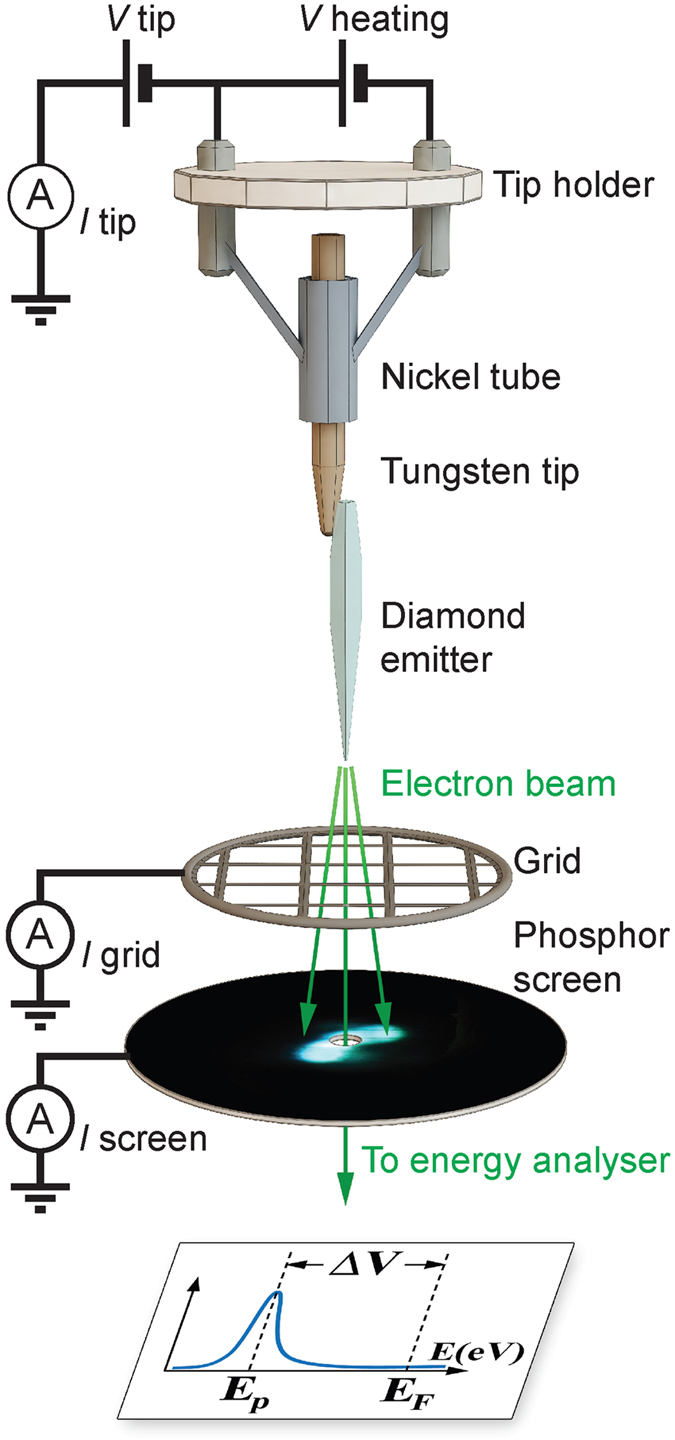
Schematic illustration of the FE experimental setup.

In this study we present results obtained for two diamond needles (Fig. 1) with different dimensions denoted DN1 (length *L* = 55 μm, max. width *d* = 0.85 μm) and DN2 (*L* = 100 μm, *d* = 3 μm). The diameters of the apexes of both needles were less than 50 nm. FE current *I* and voltage drops Δ*V* in the diamond needles as functions of time, extraction voltage *V* and temperature *T* were measured in an ultrahigh vacuum (UHV) system equipped with an electron energy analyzer (Fig. 2).

**Figure 3:**
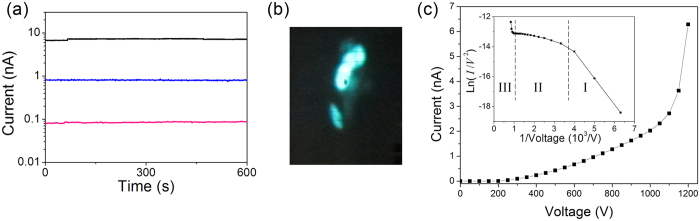
Field emission characteristics of diamond needles at room temperature. (**a**) Emission current stability of DN2 for different voltages. (**b**) FE pattern of DN1 consisting of several bright spots originating from local emission zones on the needle apex. (**c**) Current-voltage curve for DN2 at room temperature. Inset: Fowler-Nordheim plot for the same *I-V* curve. One can distinguish three regions: region I-linear dependence corresponding to standard FN tunneling, region II-saturation of current due to limited carrier supply in the depletion zone, region III-rapid current increase caused by impact ionization in the depletion zone.

Before FE measurements, the diamond needles were heated to 900 K for 30 sec in order to remove volatile molecules that usually adsorb on the surface during exposure to air. After this preliminary heat cycle, the FE current was very stable in time as demonstrated in Fig. 3a which shows the dependence of the emission current on time for three values of voltages at room temperature. FE patterns of the diamond needles consisted of several bright spots (Fig. 3b) originating from local emission zones at the needle apex.

Typical example of the FE current-voltage (*I*-*V*) curve measured for the diamond needle ND2 is shown in Fig. 3c. The curve had no hysteresis and was reproducible during subsequent voltage cycles. These can be compared with FE *I-V* curves from metal emitters which follow the well-known standard FN equation: *I* = *C*_1_(*βV*)^2^ exp(−*C*_2_*φ*^3/2^/(*βV*)), where *C*_1_ and *C*_2_ are roughly constants, *φ* is the work function and *β* is the geometrical field enhancement factor^17^. The FN plot, i.e. dependence of Ln(*I*/*V*^2^) versus 1/*V*, should give a straight line for an emitter with metallic type of conduction. For the diamond needles the FN plot is straight only for a very small current range at low voltage in region I (see inset in Fig. 3c). At higher voltage (region II) the current deviates downward from the linear dependence, in other words it ≪saturates≫. This saturation is predicted by the basic theory of FE from semiconductors^16^. Similar behavior was also observed experimentally for properly prepared semiconducting FE tips^18^,^19^,^20^,^21^ and most recently for Si^22^ and SiC^23^,^24^ nanowires. According to Baskin^16^ in p-type (as well as highly resistive n-type) semiconductors a highly resistive region (depletion zone) is formed near the emitter apex associated with the penetration of electric field. The depletion zone limits the current as in a reverse biased diode resulting in a saturation region in *I-V* curve. The theory predicts strong dependence of the current on temperature in the region II, since the thermal generation of free carriers in the depletion zone strongly affects carriers supply to the emitter apex. In the region III the electric field in the depletion zone is enough for impact ionization and the current increases rapidly.

**Figure 4:**
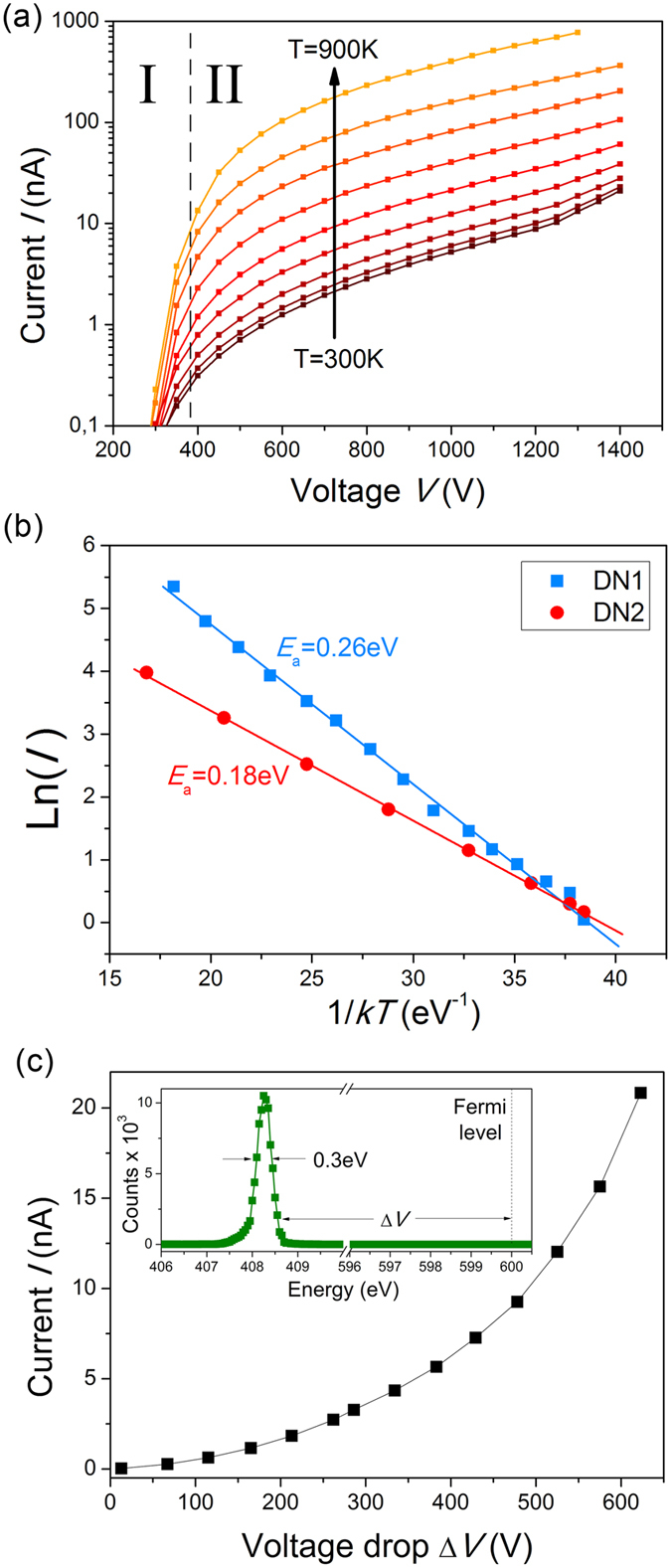
Temperature dependence of field emission from diamond needles. (**a**) *I-V* curves for DN2 measured at different temperatures in semi-log coordinates. (**b**) Arrhenius plots of the emission current for two diamond needles DN1 and DN2 at 700 V. Solid lines are linear approximations of the experimental points. (**c**) FE current versus voltage drop along the diamond needle (DN2) at room temperature. Inset shows electron energy spectrum at 1 nA.

We observed the increase of the emission current on two orders of magnitude with *in situ* diamond needles heating from 300 K to 900 K as it shown in Fig. 4a. In agreement with the theory, FE current was much less sensitive to temperature in region I than in saturation region II (Fig. 4a). The dependence of FE current on temperature in region II followed Arrhenius law *I* ~ exp(−*E*_a_/*kT*), where *E*_a_ is an activation energy and *k* is the Boltzmann constant. At constant voltage both diamond needles demonstrated linear Arrhenius plots, i.e. the dependence of Ln(*I*) on 1/*kT* with the slope equal to *E*_a_ (Fig. 4b). In the middle of the region II at 700 V the values of *E*_a_ were 0.26 eV and 0.18 eV for DN1 and DN2 correspondingly. *E*_a_ slightly decreased with increase of the voltage. The total difference of this characteristic energy at the end and beginning of the region II was about Δ*E*_a_ ~ 0.01 eV.

Together with the FE current the total electron energy distribution (TED) was measured. For both samples the TED consisted of a single peak shifted from the Fermi level to lower energies (See inset in Fig. 4c). The distance between the peak position and the Fermi level gives the voltage drop Δ*V* along the needle. Plotting *I* versus Δ*V* is equivalent to a two point measurement of transport current-voltage dependency with one bias direction available^25^. Figure 4c shows, for example, *I*(Δ*V*) dependency measured for DN2 at room temperature. The exponential behavior of *I*(Δ*V*) indicates that the electron transport mechanism in the diamond needle is different from a simple Ohmic conduction. The voltage drops reached quite high absolute values up to 700 V in the end of the region II. At constant Δ*V* the current showed an Arrhenius law exponential dependence on temperature.

## Discussion

**Figure 5:**
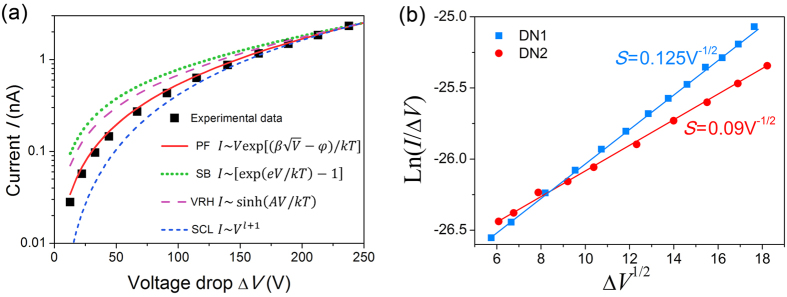
Analysis of transport mechanism of diamond needles. (**a**) Current versus voltage drop for DN2 in semi-log coordinates at room temperature and fits with Schottky barrier (SB), Poole–Frenkel (PB), variable range hopping (VRH) and space-charge-limited conduction (SCL) models. Poole–Frenkel model (solid line) gives the best fit to the experimental data. (**b**) Poole-Frenkel plots for two diamond needles DN1 and DN2. Solid lines are linear approximations of the experimental data.

The measurements show that these CVD diamond needles demonstrate stable FE currents up to 1 μA despite low electrical conductivity expected for the wide band gap material. This electrical conduction of the diamond needles could be explained by the accidental doping during synthesis process. The possible dopants are silicon diffusing from the substrate material during CVD process and nitrogen originating from the gaseous environment. The presence of nitrogen and silicon centers in the diamond needles was previously confirmed by photo- and cathodo-luminescence experiments^26^, while the presence of other dopants seems impossible because of experimental conditions and independent confirmation by EDX measurements. However the positions of the energy levels of the defects assigned to nitrogen and silicon impurities^27^ relative to the diamond conduction band are well above 1 eV which is considerably higher than obtained values of activation energy *E*_a_ below 0.3 eV. A more likely transport mechanism is trap-assisted conduction with characteristic trap energy *E*_a_. The trap-assisted mechanism can also explain the non-linear behavior of the two-point current-voltage curve observed in experiment (Fig. 4c). However, one should take into account that the current can be limited in the contact region between the tungsten tip holder and the diamond needle. There are several possible mechanisms of bulk conduction in dielectrics (hopping conduction, Poole-Frenkel mechanism, space-charge-limited conduction etc.) as well as contact limited conduction (Schottky emission, Fowler-Nordhem tunneling etc.)^28^. Following the analysis performed by Choueib^24^ we performed fits of *I*(Δ*V*) curves with four different possible models: Schottky barrier (SB), Poole–Frenkel (PF), space-charge limited conduction (SCL) and variable range hopping (VRH). The fits and corresponding formulae are shown in Fig. 5a for the experimental *I*(Δ*V*) data from Fig. 4c in semi-log coordinates. The best fit was given by PF mechanism consisting in thermal excitation of trapped electrons into the conduction band, which is enhanced by the reduction of the trap barrier height by electric field. It is worth to note that PF conduction was observed previously for thin CVD diamond films, for example, by Göhl^9^.

The current dependence on applied voltage due to the PF mechanism is described by^28^:


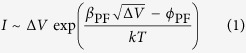


where *ϕ*_PF_ is the barrier height of the trap potential and *ß*_PF_ is the barrier lowering coefficient which is given by:


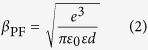


where *e* is electron charge, *ε*_0_ is the permittivity of free space, *ε* is dielectric constant of the material and *d* is the sample’s length.

According to equation (1) *I(*Δ*V)* curve of PF conduction should follow a linear dependence if plotted in PF coordinates, i.e. Ln(*I*/Δ*V)* versus Δ*V*^1/2^ with slope given by:


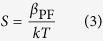


Using equation 3 and 2 the value of dielectric constant *ε* can be determined from the fit of the PF plot. Both diamond needles demonstrated linear PF plots (Fig. 5b) with slopes of *S*_DN1_ = 0.125 and *S*_DN2_ = 0.09 V^−1/2^.

Dielectric constants derived from the slopes are of *ε*_DN1_ = 9.2 and *ε*_DN2_ = 9.6. The fact that obtained values are close to each other for the two diamond needles with different dimensions supports the validity of the performed analysis and indicates that the electric characteristics of the needles does not vary significantly. However, dielectric constant of bulk diamond *ε*_bluk_ = 5.7 is much lower than the values obtained from the above analysis. Previous investigations by TEM^14^ have shown that the core of the needles has almost perfect single-crystal diamond structure and therefore should have dielectric constant close to *ε*_bluk_. At the same time the needles contain various defects (twins, stacking faults etc.) which are concentrated in the pre-surface region and do not propagate into the middle of the crystal. Therefore we suppose that when the voltage is applied to the needle most of the current is flowing due to PF mechanism through the surface region where the concentration of traps is higher. Thus effective dielectric constant which is obtained from the analysis of *I-V* curve can be higher due to the surface effect. Another reason for the discrepancy of dielectric constant values may be the simplicity of the used conduction model which does not take into account a complex shape of the emitter and nonhomogeneous distribution of electric field inside the needle, however such analysis is out of the scope of the current study.

In summary, the single-crystal diamond needles with high aspect ratio produced by thermal oxidation of polycrystalline CVD diamond films were examined in the FE setup equipped with an electron energy analyzer. The current-voltage dependencies demonstrated pronounced saturation, largely deviating from Fowler-Nordheim law, which was explained by the limitation of the emission current in the highly resistive depletion zone formed due to penetration of the electric field into diamond. The current in the saturation region was sensitive to temperature and increased by two orders of magnitude upon raising the temperature from 300 K to 900 K. The electron energy distributions exhibited a single energy peak, shifted from the Fermi level by the voltage drop along the needle. The dependence of the voltage drop on emission current was analyzed in comparison with different transport mechanism models. The Poole-Frenkel conduction model gave the best fit to experimental data with characteristic trap energy in the range of 0.2–0.3 eV.

The fundamental study reported here improves our understanding of diamond electron emission and its surface properties, and can thus serve as a base for understanding the other characteristics of diamond electronic devices. The diamond needles demonstrated excellent emission stability, thermal sensitivity and saturation phenomenon which open different ways of their application in the heat-controlled current sources and sensors. Geometrical shape with small apex size allows usage of the diamond needles as stable point source of electrons.

## Methods

### Samples preparation

Polycrystalline diamond films were produced by chemical vapor deposition (CVD) on silicon substrates from hydrogen–methane gas mixture activated by a direct current discharge. The textured films consisting of partially oriented crystallites were obtained with appropriate choice of the CVD process parameters. A notable feature of the films is their face side containing square (001) diamond facets surrounded by nano-diamond and disordered carbon material. The CVD films were then oxidized by exposure to normal air atmosphere at about 900 K for several hours. As a result the less ordered (nano-diamond and disordered carbon) component of the film was oxidized and eliminated from the film due to gasification. The largest fraction of the film in the form of needle-like diamond crystallites remained on the substrate because of their much higher stability to oxidation. The crystallites have geometrically perfect shape of rectangular pyramid as identified with optical and scanning electron microscopy (SEM)^13^. The needles, obtained by this method, are represented by perfect undisturbed diamond crystallites, which is evidenced by the Large Area Convergent Beam Electron Diffraction (LACBED) pattern with straight and sharp High-Order Laue-Zone (HOLZ) diffraction lines^14^ which could not appear in defective crystal^29^. Other details on the synthesis of diamond needles and characterization procedures can be found elsewhere^13^.

Individual needles were attached to the ends of tungsten tips using SEM instrument equipped with a micromanipulator. Platinum deposition by means of the gas injection system was used to provide fixation of the needles on the macroscopically large tungsten holder.

### Field emission

FE measurements were performed in an ultrahigh vacuum system at pressure of 4 × 10^−10^ Torr (Fig. 2). The tungsten tip with a diamond needle was held in a nickel tube welded to a tantalum loop in order to control the temperature of the sample *in situ* by Joule heating. DC voltage was applied between the tungsten tip and an extractor grid mounted 2 mm away from the diamond needle apex and perpendicular to its axis. The electrons emitted from the needle passed through the grid and formed the FE pattern on a phosphor screen situated 2 cm away from the grid. A part of the emitted electrons could pass through a 1 mm probe hole in the screen and were collected by an electron energy analyzer. The total electron energy distribution detected with the analyzer consisted of a single peak shifted from the Fermi energy to a value corresponding to the voltage drop Δ*V* in the diamond needle.

## Additional Information

**How to cite this article**: Kleshch, V. I. *et al.* Single Crystal Diamond Needle as Point Electron Source. *Sci. Rep.* **6**, 35260; doi: 10.1038/srep35260 (2016).
